# Exploring the boundary between a siphon and barometer in a hypobaric chamber

**DOI:** 10.1038/srep04741

**Published:** 2014-04-22

**Authors:** Stephen Hughes, Som Gurung

**Affiliations:** 1 Department of Chemistry, Physics and Mechanical Engineering, Queensland University of Technology, Brisbane, Queensland 4001, Australia; 2 Paro College of Education, Royal University of Bhutan, Paro, Bhutan and Department of Chemistry, Physics and Mechanical Engineering, Queensland University of Technology, Brisbane, Queensland 4001, Australia

## Abstract

Siphons have been used since ancient times, but exactly how they work is still a matter of debate. In order to elucidate the *modus operandi* of a siphon, a 1.5 m high siphon was set up in a hypobaric chamber to explore siphon behaviour in a low-pressure environment. When the pressure in the chamber was reduced to about 0.18 atmospheres, a curious waterfall-like feature appeared downstream from the apex of the siphon. A hypothesis is presented to explain the waterfall phenomenon. When the pressure was reduced further the siphon broke into two columns - in effect becoming two back-to-back barometers. This experiment demonstrates the role of atmospheric pressure in explaining the hydrostatic characteristics of a siphon and the role of molecular cohesion in explaining the hydrodynamic aspects.

The first recorded use of the siphon was in ancient Egypt where the Egyptians used siphons in irrigation and winemaking to separate wine from the lees^1^,^2^. Today, siphons are used in the cotton industry to transfer water from canals to irrigation channels. Over the last two decades siphonic drainage has become popular, enabling large volumes of water to be removed from buildings with a large roof area (for example the Olympic stadium in Sydney).

Over the last few years there has been controversy over how siphons work^3^,^4^,^5^,^6^,^7^,^8^,^9^. Two competing models have emerged. In one model, water flowing out of a siphon generates a low-pressure region at the crown so that atmospheric pressure pushes water into the siphon. In another, the weight of water flowing out of a siphon pulls water into the siphon via liquid cohesion.

The siphon debate has also had an impact in the field of botany in relation to how water can rise above the 10 m siphon limit in trees^10^. This implies some kind of continuous link between water entering the roots and transpiring through the leaves. In the field of biomedicine there is controversy over whether the siphon principle operates in the human and other circulations^11^.

An argument often used in support of the atmospheric model of the siphon is the fact that the maximum height of a siphon is almost the same as a barometer. The experiment described in this paper explores the boundary between the siphon and barometer.

## Results

In the first run there was little change in flow until the siphon reached 25 000 feet (37.60 kPa, 0.37 atm), when the siphon became choked with bubbles and stopped. The chamber was opened and adjustments made to the siphon, which included putting a piece of half tubing beneath the crown of the siphon to smooth the curve at the top. In the second run, the ascent was slowed to allow outgassed CO_2_ to dissipate. This enabled the siphon to reach 35 000 feet (23.84 kPa, 0.23 atm) without any bubbles appearing. Above 35 000 feet, the ascent was performed in 1 000 feet increments. About one minute was allowed at each ‘stop' for outgassing.

Between 39 000 (19.67 kPa, 0.19 atm) and 40 000 feet (18.75 kPa, 0.18 atm) a curious phenomenon was observed – a waterfall appeared just beyond the apex, as seen in one of the supplementary video clips. The effect was just noticeable at 39 000 feet and was at a maximum at 40 000 feet. We have called this feature a waterfall since it looks like a waterfall with water falling over an edge and plunging into a pool.

Sequences of video data with flow values one second apart were used to calculate the siphon flow at strategic moments. At least 10 values were used to calculate the mean and standard deviation. The following values are the mean flow ± the standard deviation. The flow at the beginning of the main experimental ‘flight' at sea level was 5.6 ± 0.05 l min^−1^, at the end of the experiment, back at sea level, the flow was 5.32 ± 0.02 l min^−1^. At 39 000 flow was 5.24 ± 0.07 l min^−1^ and at 40 000 feet during operation of the waterfall, flow was 4.11 ± 0.10 l min^−1^.

Figure 3 shows a plot of siphon flow at 39 000 feet and during ascension to 40 000 feet over a period of about a minute. Flow reduced by about 20% but then recovered slightly. There was increased turbulence when the waterfall was in operation, which decreased flow slightly as expected, however the natural feedback system caused the flow to recover slightly before stabilizing.

Measurements from the video of the waterfall indicate that the height of the waterfall was 41 ± 1 cm, about the same as the height difference between the upper and lower reservoir levels. Between 40 000 and 41 000 feet (17.87 kPa, 0.17 atm) the waterfall stopped and the siphon split into two columns, with the ascending column being just below the apex and the descending column at about the same place as the waterfall pool (see supplementary video ‘Stopped at 41000 feet'). (When the siphon stopped, power was cut to the return pump to prevent the lower reservoir from running dry). When the altitude was decreased from 40 000 to 39 000 feet (see video) the level of the ‘pool' of the waterfall gradually rose to meet the edge, and by 39 000 feet the waterfall had more or less disappeared. After the siphon had stopped, when the altitude was decreased the two separate columns of water rose and re connected and the siphon restarted.

## Discussion

Apart from the issues with the bubbles, flow remained more or less constant during ascension indicating that siphon flow is independent of ambient barometric pressure, at least until the pressure becomes low enough to cause cavitation. In this experiment cavitation began to occur at 39 000 feet and between 40 000 and 41 000 feet the siphon broke and separated into two columns with a height difference of ~40 cm. Between the first signs of cavitation and total separation of the column the siphon continued to flow via the waterfall phenomenon (figure 4). The fact that the height of the waterfall was approximately the same as the height difference between the upper and lower reservoir levels (bearing in mind that the height difference would have varied slightly throughout the experiment), suggests that the siphon was part siphon and part barometer. When flow stopped the siphon became two back-to-back barometers.

The waterfall phenomenon was caused by water boiling at the apex of the siphon. At an altitude of 40 000 feet, air pressure is sufficient to support a column of water 1.86 m high. At an altitude of 40 000 feet, the pressure at the apex of the siphon is equivalent to the pressure at 78 000 feet, which explains why the siphon stopped working at a height 0.34 m less than 1.86 m.

The pressure in a siphon above the level of the upper reservoir is always less than the ambient atmospheric pressure in accordance with the Bernoulli equation 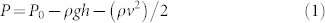
where *P* is the pressure at a point in the siphon at a height *h* above the level of the upper reservoir, *P*_0_ is the ambient atmospheric pressure, *ρ* the density of the water in the siphon, *g* the acceleration due to gravity and *v* the average flow velocity in the siphon.

Atmospheric pressure at a given altitude can be found using various calculators. For this experiment the 1976 Standard Atmosphere Calculator^12^,^13^ was used. The vapour pressure of water (*P_v_*) as a function of temperature (*T*) in °C is given by the Antoine equation: log_10_*P_v_* = *A* − *B*/(*C* + *T*) where *A*, *B* and *C* are constants, which for water are 8.07131, 1730.63 and 233.426 respectively. The vapour pressure of water at 25 °C (the temperature at which this experiment was conducted) is 23.68 mmHg (3.15 kPa, 0.03 atm). An equation for the maximum height (*h_m_*) of a siphon can be derived by equating the pressure in the siphon to the vapour pressure (*P_v_*) of water at a given temperature between zero and 100 °C 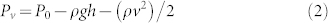

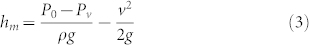
When *v* = 0, the equation gives the maximum height of a barometer, and when *v* > 0, the maximum height of a siphon. In this experiment, the flow rate of the water in the siphon at 40 000 feet was about 4 l min^−1^. For an internal tube diameter of 12 mm, this equates to an average velocity of 0.59 m s^−1^. At sea level, the maximum height of a siphon at 25 °C with a flow velocity of 0.59 m s^−1^ is 10.0 m. At 40 000 feet the maximum height is 1.57 m, and at 41 000 feet, 1.48 m. In this experiment, the siphon stopped operating between 40 000 and 41 000 feet, which accords well with theory since 1.52 m lies between 1.48 and 1.57 m.

The flow of the water in the tube reduces the fluid pressure by an amount equal to 1/2*ρv*^2^. For a velocity of 0.59 m s^−1^, this gives a pressure reduction of 0.174 kPa, equivalent to 1.3 mmHg or 1.77 cm of water. As can be ascertained by viewing the video of the waterfall, the falling column of water is a mixture of water and water vapour. This mixture falls over the edge and drops into the lower column of water, which acts like a pool of water. The water looks like it is in freefall.

When the waterfall was in operation, there was no continuous column of water between the inlet and outlet and yet the siphon appeared to operate normally with only a slight reduction in flow. (During the experiment it took a while for the waterfall to be noticed since there was no obvious decrease in flow). The height of the pool appeared to be constant while the flow was constant analogous to water from a tap pouring into a sink at the same rate as the outflow through the plughole so that the water level in the sink is constant.

It is clear that when the waterfall phenomenon appeared in the siphon, the siphon must be operating differently from normal. An important point to note is that the stability of the upper and lower reservoir levels during the operation of the siphon indicates that no energy is transferred between the siphon and atmosphere (i.e. the vector product of the force and distance is zero). In other words, the pressure of the atmosphere does not push water into the siphon, neither does the water in the lower reservoir push on the atmosphere.

However, since atmospheric pressure decreases with height, the atmosphere in the chamber would have had a very small retarding effect on the flow since the pressure above the lower reservoir was slightly greater than the upper reservoir - the pressure difference being *ρgh* where *ρ* is the density of air at the given barometric pressure, *g* the acceleration due to gravity and *h* the height difference between the upper and lower reservoir levels.

When the waterfall was in full operation, the pool level was about 40 cm below the apex, and the siphon was in effect a partial barometer. When the siphon stopped and separated into two columns, the weight of the two columns on either side of the apex decreased the pressure at the apex by an amount equal to *ρgh*. The two columns of water were in effect two back-to-back syringes pulling on the space between containing mostly water vapour. The difference in height between the two columns was equal to the difference in height between the buckets, i.e. 40 cm.

When the altitude was decreased from 40 000 to 39 000, the waterfall gradually closed. The difference in pressure between 40 000 and 39 000 is 0.924 kPa, equivalent to 0.9 m of water, more than enough to close the 40 cm gap. An interesting aspect of the closure of the waterfall is that as the pressure was increased, resulting in closure of the waterfall, flow remained constant. From this it can be inferred that an increase in ambient pressure does not have any effect on the ascending section of a siphon.

A key question is how water is raised from the inlet to the top of the siphon when the waterfall is in operation. If an experiment is performed with a simple ‘kitchen' barometer, for example, a straw pushed into the water and then lifted with a finger placed over the end, when water level in the glass is varied, the level of the water in the straw remains constant. This demonstrates that although atmosphere pressure holds the ascending column in balance it cannot push water into the inlet of a siphon.

From the relevant supplementary video it can be seen that the waterfall begins just downstream from the apex of the siphon. There appears to be a tongue of water a few cm in length just downstream from the apex of the siphon (the known diameter of the tube can be used as a scale). Although the tongue of water appears to be mixed with bubbles it is otherwise intact.

An interesting question is why the edge of the waterfall appears just downstream of the apex of the siphon? Since the lowest pressure in a siphon occurs at the top, we would expect cavitation to occur at the apex and immediately break the column of water. One possibility to explain this phenomenon is that although cavitation occurs at the apex, due to the velocity of the water, bubbles do not expand sufficiently to break the circulation until downstream of the apex.

An hypothesis proposed here is that the weight of the tongue of water falling over the edge of the waterfall is sufficient to suck water into the siphon and maintain flow comparable to the normal operation of the siphon. This hypothesis presupposes a link between water in the waterfall and the inlet of the siphon with the column of water effectively acting like a chain. In essence the waterfall is like a mini siphon but with a key difference being that the outflow of the siphon is just downstream from the apex.

In a normal siphon, the height of the outflow, or the height of the water in the lower reservoir, must be lower than the top surface of the upper reservoir. However, when the waterfall is in operation, the outlet of the siphon is actually higher than the inflow. The siphon has in effect become a combined siphon and barometer. The ascending section is a siphon with an outflow higher than the inflow, and the descending section a barometer, albeit a dynamic barometer, since water flowing out the bottom is replaced by water dropping into the top.

To test the hypothesis we can estimate the length of the column of water downstream from the apex (Δ*h*) required to drive flow through the ascending portion of the siphon at the measured flow rate. We will assume that water approximates a Newtonian fluid, is incompressible, not accelerating and flow is laminar. The Reynolds number (*Re*) for the experimental setup is 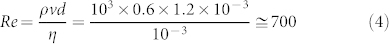
where *ρ* and *v* are as previously defined and the characteristic length (*d*) is taken as the diameter of the tube, since the tube is horizontal at the top of the siphon. Observation of the bubbles indicated that flow was reasonably lamina - i.e. the bubbles did not travel in vortices characteristic of turbulent flow.

In view of this, it seems reasonable to apply the Poiseuille equation to estimate the length of the water column beyond the apex of the siphon required to generate the same flow as the intact siphon. The vertical column of water in the ascending section of the siphon is supported by the atmosphere and so effectively is like a horizontal tube. Therefore the pressure gradient driving flow in the ascending section of the siphon when the waterfall is in operation is equal to the pressure (*P*) generated by the column of water of height Δ*h* falling over the edge of the waterfall, i.e. 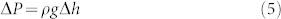
The Poiseuille equation (6) gives the flow (Q) of a viscous fluid through tube of circular cross-section for a given pressure gradient (Δ*P*). 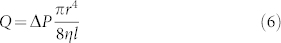
where *r* is the radius of the tube, *l* the length of the tube and *η* viscosity (the value for water taken as 10^−3^ Pa s). Substituting the expression for Δ*P* in equation (5) into (6) above and rearranging gives 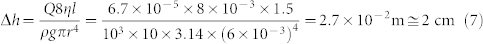
This value accords with the length of the tongue of water observed in the video and therefore the hypothesis seems reasonable. Studies have shown that water can exhibit a high tensile strength and therefore a tube of water this length is easily able to pull up the vertical column of water^14^,^15^,^16^,^17^.

The siphon waterfall has similarities with what we might call the *flush siphon* as shown in figure 5. This type of siphon is used in some toilet flush systems and built into canal walls to prevent breaching. For example, this type of siphon has been built into the walls of the Canal du Midi in southern France to prevent breaching of the canal wall during heavy rainfall. The principle of the flush siphon is also used in a Pythagoras cup, which contains a central column siphon that drains drink from the cup if the cup is filled above the top of the siphon.

In a flush siphon, as the water level external to the siphon rises, so the level in the ascending section of the siphon rises until it reaches the apex. In the ascending section of the siphon, the column of water is effectively weightless as it is supported by the water external to the tube and only a small amount of energy sufficient to overcome friction is required to raise it. When the water column passes over the apex the small amount just downstream from the apex is sufficient to pull water into the inlet of the siphon. An important point to note is that the flush siphon will continue to flush even when the water level external to the ascending tube is lower. In the waterfall siphon the column of water passing over the apex cannot drop below a certain level due to cavitation. When cavitation occurs, the water column breaks and is in free fall until plunging into the pool at the top of the barometer section.

In the case of the constant flow siphon used in this experiment, it might be argued that although the upper and lower reservoir levels are stable and therefore atmospheric pressure is not pushing water into the siphon, the whole system is like a pressurized tank so that the water flowing into the upper reservoir pushes water into the siphon.

An interesting question is what effect the return flow has on the siphon. If the end of the return circulation were directed into siphon inlet, the extra pressure would act like a pump and augment atmospheric pressure so the siphon would stop working at a lower ambient pressure, or conversely stop working at a greater height if the height of the siphon could be raised. We would still have to explain how water tipped over the edge of the waterfall.

In this experiment the siphon tube rose vertically out of the bucket whereas the return tube was direction downwards on the opposite side of the bucket to the inlet. Therefore any pressure enhancement effect would have been small, which is borne out by the fact that siphon stopped working at the expected ambient pressure. The general effect of the return flow is to slightly perturb the surface of the water in upper reservoir. A small rise in the surface around the ascending siphon tube would increase flow slightly and a small drop decrease flow. The perturbations would average out at a certain level. Once again this effect would have been very small since siphon flow was stable.

**Figure 1:**
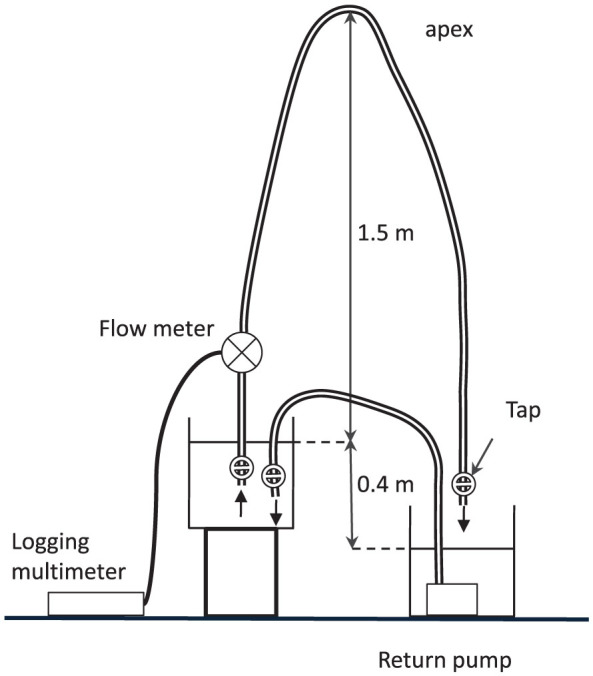
Schematic diagram of the siphon constructed in the hypobaric chamber.

A continuous flow siphon can be set up (as in figure 1) in which the return pump is switched off and then on again. When the pump is switched off the water level in the upper reservoir falls and siphon flow reduces. When the pump is switched back on, the water level in the upper reservoir rises and siphon flow increases.

The essential point here is that at any given level in the upper reservoir, siphon flow is the same whether or not the pump is switched on and the level is rising, or switched off and the level falling. Therefore, siphon flow is independent of flow into or out of the upper reservoir and only dependent on the water level. The same experiment can be performed using the apparatus in figure 5 in which the inflow can be adjusted by turning a water faucet on and off.

It follows from the above analysis that there must be a direct cohesive connection between water molecules flowing in and out of a siphon. This is true at all atmospheric pressures in which the pressure in the apex of the siphon is above the vapour pressure of water, an exception being ionic liquids. Boatwright et al.^18^ describe the operation of an ionic liquid siphon in a high vacuum [5 × 10^−9^ Pa].

It would be interesting to repeat the experiment described in this paper with an ionic liquid. In principle an ionic liquid siphon should operate in an ultra-high vacuum environment, for example on the Moon and also operate at a much greater height than 10 m since the water will not boil. A lunar siphon would also have a slightly greater flow in the absence of an atmosphere for the reasons cited above.

In conclusion, this experiment has explored the boundary between the barometer and siphon and demonstrates that atmospheric pressure is able to explain the hydrostatic characteristics of a siphon and molecular cohesion the hydrodynamic features.

## Methods

**Figure 2:**
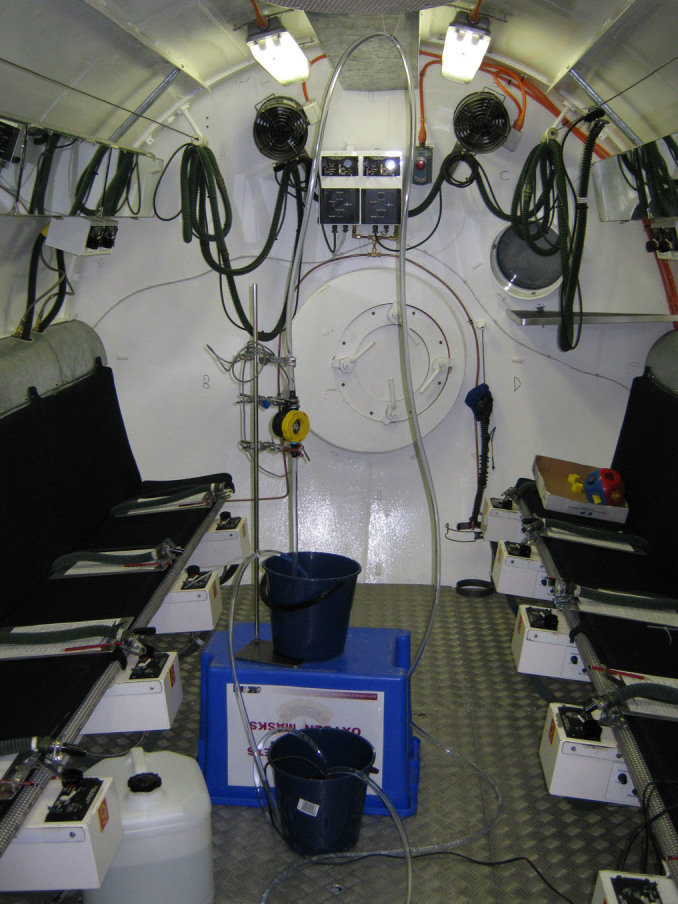
Photo of siphon setup in the hypobaric chamber.

**Figure 3:**
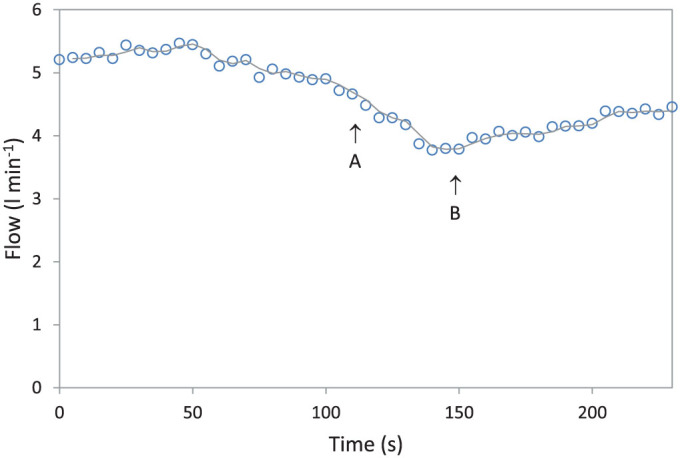
Plot of flow versus time with the siphon at 39 000 feet and then at 40 000 feet with the waterfall present. The pressure in the chamber was reduced between points A and B over a period of about a minute. Flow reduced but then recovered slightly.

**Figure 4:**
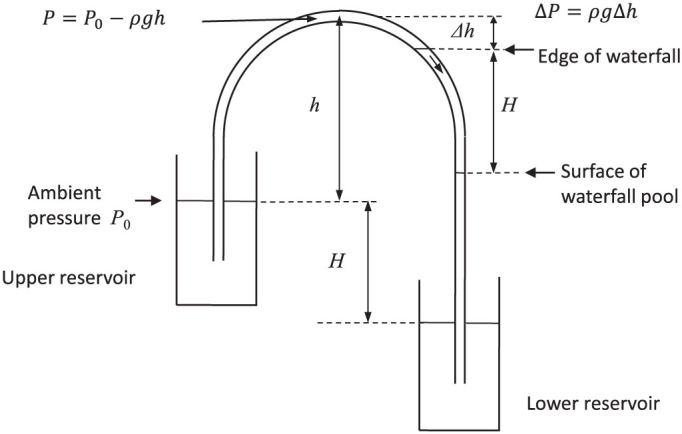
Schematic diagram of the siphon at the boundary between a siphon and barometer with the waterfall in operation. A particular feature to note is that the height of the waterfall (*H*) is approximately the same as the height difference between the upper and lower reservoir water levels.

**Figure 5:**
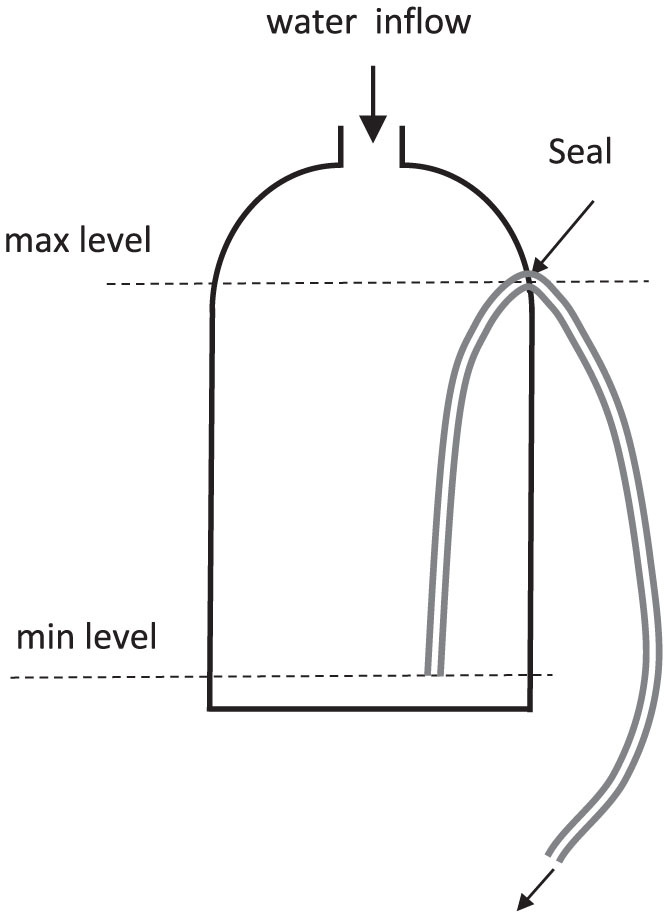
Schematic diagram illustrating the principle of operation of a flush siphon in which the siphon operates when the water level in the container reaches the apex of the tube. (A video of this type of siphon operating is available in supplementary materials).

A siphon was set up in a hypobaric chamber at the Royal Australian Air Force (RAAF) Institute of Aviation Medicine (AVMED), located at RAAF Base Edinburgh, north of Adelaide in South Australia (SA). The experimental arrangement is shown schematically in figure 1 and photographically in figure 2. The upper and lower reservoirs were identical 9-litre plastic buckets obtained from a local hardware store. The siphon was constructed from 12 mm internal diameter (ID) PVC tubing obtained from an irrigation company. A flow meter (Flow Stat ES/Economy, Lake Monitors Inc, Milwaukee, WI 53215) with pulse output was inserted into the ascending section of the siphon and plastic cable ties used to attach the top of the siphon to a grill on the ceiling of the chamber. The flow meter was connected to a 12 VDC power supply that could be switched on and off from outside the chamber. The flow meter was connected to an Agilent 1253A logging multimeter set to frequency mode. Frequency values were displayed on the screen once per second and every fifth data point stored in the internal memory.

The multimeter was oriented so a video camera could record the display panel via a porthole in the side of the hypobaric chamber at significant moments during the experiment. The Agilent meter contained a built in temperature probe and the meter temperature reading was taken as the temperature of the water since the water had been in the chamber for about 24 hours prior to the experiment. Throughout the three hours of the experiment, the temperature of the chamber was 25 ± 1°C. The outlet of the siphon tube was directed into a bucket placed on the floor of the hypobaric chamber. Retort clamps attached to a heavy duty retort stand (specially manufactured for the experiment by Henstock Technologies, Lobethal, SA) were used to support the flow meter.

Plastic irrigation taps were placed at either end of the siphon tube. A small pump capable of maintaining a flow head of 1.2 m was placed in the lower reservoir (figure 2) and water returned to the upper reservoir via a length of 12 mm ID PVC tubing. A tap was placed on the end of the return tube to control flow into the upper reservoir of the siphon.

A twenty litre container of distilled water, acquired from a company in Adelaide, was placed in the hypobaric chamber for 30 minutes at a pressure equivalent to an altitude of 60 000 feet (7.17 kPa, 0.07 atm) to remove carbon dioxide. However, the main experiment commenced two and half hours after degassing and therefore some air would have dissolved in the water during this period.

To prime the siphon, the tubing was taken to a sink outside the hypobaric chamber and filled with the degassed water, care being taken to remove all bubbles. The siphon tubing was taken back to the chamber and the siphon set up as shown in figures 1 and 2. Both taps at either end of the siphon were opened in quick succession to start the flow and then the pump was switched on. The tap at the end of the return tube was adjusted until the return flow was approximately the same as the siphon flow.

The return flow did not have to exactly match the siphon flow since a natural feedback system was in operation. If the return flow was slightly lower than the siphon flow, the water level in the upper reservoir reduced, decreasing the siphon height, which in turn decreased siphon flow. After a minute or so the inflow and outflow equilibrated and the upper and lower reservoir levels remained constant. This enabled stable siphon flow to be maintained for several hours, which was necessary since the equipment was inaccessible when the chamber was de pressurised. Another advantage of the siphon-pump circulation is that stable, non-pulsatile flow can be maintained for several hours.

Prior to closing the chamber, the siphon height, the vertical distance between the water level in the upper reservoir and top of the siphon, was measured as 152 ± 1 cm. The vertical height between the upper and lower reservoirs was 40 cm ± 1 cm. While the experiment was running, the actual height difference was unknown and would have varied slightly as the flow changed. When the siphon was running stably the chamber was closed and depressurisation commenced.

One of the AVMED staff ‘piloted' the chamber whilst another read off the altitude in feet. The ‘flight deck' had three pressure sensors/altimeters in operation. The first was a Druck DPI 740 precision pressure digital indicator with an accuracy of ±0.004 inHg (±0.0135 kPa), calibrated every 12 months by Thermo Fisher Scientific Pty Ltd, Melbourne, VIC, Australia. The display on this unit was set to convert barometric pressure to altitude above sea level in feet. The second was a Servo Altimeter. The inverter voltage powering this altimeter is checked every 12 months during routine chamber servicing by the Hyperbaric Chamber Technicians. The third was a mechanical altimeter. All three must agree within a certain pressure. If this is not the case a fault condition is reported and acted on. At several points throughout the experiment the multimeter display was videoed and the information used to match events in the experiment with the data recorded in the internal memory of the meter.

## Author Contributions

S.H. Wrote the paper and performed the experiments at the Edinburgh Royal Australian Air Force base and S.G. assisted with the flush siphon experiments in the laboratory at Queensland University of Technology. Both authors have read the paper.

## Supplementary Material

Supplementary Information Flush siphon

Supplementary Information Walking into chamber

Supplementary Information Water fall at 40000 feet

Supplementary Information From 40000 to 39000 feet closure of the waterfall

Supplementary Information Stopped at 41000 feet

Supplementary Information Recovered siphon at 30000 feet
